# Intracellular product recycling in high succinic acid producing yeast at low pH

**DOI:** 10.1186/s12934-017-0702-0

**Published:** 2017-05-23

**Authors:** S. Aljoscha Wahl, Cristina Bernal Martinez, Zheng Zhao, Walter M. van Gulik, Mickel L. A. Jansen

**Affiliations:** 1 Department of Biotechnology, Delft University of Technology, Van der Maasweg 9, 2629 HZ Delft, The Netherlands; 2 Applikon Biotechnology B.V., Heertjeslaan 2, 2629 JG Delft, The Netherlands; 3 DSM Biotechnology Center, Alexander Fleminglaan 1, 2613 AX Delft, The Netherlands

**Keywords:** Metabolic flux analysis, *Saccharomyces cerevisiae*, ^13^C labeling, Succinic acid production

## Abstract

**Background:**

The metabolic engineering of *Saccharomyces cerevisiae* for the production of succinic acid has progressed dramatically, and a series of high-producing hosts are available. At low cultivation pH and high titers, the product transport can become bidirectional, i.e. the acid is reentering the cell and is again exported or even catabolized. Here, a quantitative approach for the identification of product recycling fluxes is developed.

**Results:**

The metabolic flux distributions at two time-points of the fermentation process were analyzed. ^13^C labeled succinic acid was added to the extracellular space and intracellular enrichments were measured and subsequently used for the estimation of metabolic fluxes. The labeling was introduced by a labeling switch experiment, leading to an immediate labeling of about 85% of the acid while keeping the total acid concentration constant. Within 100 s significant labeling enrichment of the TCA cycle intermediates fumarate, iso-citrate and α-ketoglutarate was observed, while no labeling was detected for malate and citrate. These findings suggest that succinic acid is rapidly exchanged over the cellular membrane and enters the oxidative TCA cycle. Remarkably, in the oxidative direction malate ^13^C enrichment was not detected, indicating that there is no flux going through this metabolite pool. Using flux modeling and thermodynamic assumptions on compartmentation it was concluded that malate must be predominantly cytosolic while fumarate and iso-citrate were more dominant in the mitochondria.

**Conclusions:**

Adding labeled product without changing the extracellular environment allowed to quantify intracellular metabolic fluxes under high producing conditions and identify product degradation cycles. In the specific case of succinic acid production, compartmentation was found to play a major role, i.e. the presence of metabolic activity in two different cellular compartments lead to intracellular product degradation reducing the yield. We also observed that the flux from glucose to succinic acid branches at two points in metabolism: (1) At the level of pyruvate, and (2) at cytosolic malate which was not expected.

**Electronic supplementary material:**

The online version of this article (doi:10.1186/s12934-017-0702-0) contains supplementary material, which is available to authorized users.

## Background

For several products, current microbial hosts can reach very high product titers. Examples are succinic acid, with final product concentrations reaching above 100 g/L [1], pyruvate [2], lactic acid [3] and many more. At such high titers and low pH, product transport could become bidirectional, i.e. the product is reentering the cells by back-diffusion or facilitated transport and can potentially be catabolized. Using high succinic acid production as case study, an approach for the identification of product recycling is presented. Succinic acid is an important building block for the production of the biodegradable plastic polybutylene succinate (PBS), polyester polyols, plasticizers and polyurethanes and should replace the petrochemical-derived precursor adipic acid [4]. Commercial scale production has been achieved by several consortia, nevertheless, higher production yields can further strengthen the competitiveness and broaden the application in the market, especially when the oil-price is comparably low.

Metabolic engineering for succinic acid production has focused on the expression of the reductive TCA cycle, glyoxylate cycle, and succinic acid exporters in different microbial hosts (see review articles like [1, 5, 6]). Current production strains reach yields of up to 1.74 mol (SA)/mol (Glc) [6, 7] and titers above 100 g/L, which can lead to precipitation at low pH (solubility limit is 75.5 g/L at 25 °C). Several groups have focused on *E. coli* [8–10], an organism which has some drawbacks for the performance in large-scale production vessels [1]. *E. coli* is sensitive to bacteriophage infections [11] and needs a near-neutral cultivation pH, which requires the addition of stoichiometric amounts of alkali during the fermentation process.

To circumvent the disadvantages of carrying out the fermentation at high pH, companies and research groups have engineered eukaryotic hosts like *S. cerevisiae* [12], *Yarrowia lipolytica* [13], or *Candida* strains [14]. These strains can be cultivated at acidic conditions below the pKa of succinic acid, facilitating the downstream processing (crystallization) and eliminating side products originating from alkali addition, e.g. gypsum [6, 15].

In this study, we analyze the metabolic flux distribution and putative losses due to transport as well as intracellular cycles under production conditions (fed-batch). Especially, labeled product (succinic acid) is used as tracer and modeling approaches are applied to quantify product degradation and recycling. Identification of product recycling and intracellular cycles, including putative parallel routes in different cellular compartments can further improve metabolic engineering strategies and identify relevant product and energy losses.

## Methods

### Strain

The succinic acid producing strain of *S. cerevisiae* used in this study was derived from DSM strain SUC-632, which is described in patent WO2013/004670 [16], and was obtained through classical strain improvement campaigns and the insertion of *E. coli* fumarase B (fumB, E.C. 4.2.1.2, UniProt accession number P14407). The expression of the fumB gene was controlled by the native TDH1 promoter and the TDH1 terminator. The synthetic expression cassette including appropriate restriction sites was synthesized by GenArt (Regensburg, Germany). This synthetic fragment was cloned in a vector containing a KanMX marker which allows for selection for growth in the presence of G418. The KanMX marker, flanked by lox66 and lox71 sites [37], were removed by the action of Cre-recombinase, as described by Guldender et al. [17]. The fumB cassette and the lox66-KanMX-lox71 sequences were flanked by sequences that allow integration by double cross-over at the YPRCtau3 locus, which is located on chromosome XVI. The fumB and KanMX expression cassettes flanked by YPRCtau3 were isolated from the vector by restriction enzyme digestion and were used for transformations.

Transformants were selected on yeast extract bacto peptone (YEP) 2% galactose plates supplemented with 200 µg G418/mL for selection of transformants containing the KanMX marker, yielding multiple transformants. Presence of the introduced fumB gene was confirmed by PCR.

### Cultivation conditions

#### Seed pre-culture conditions

The seed-culture was prepared in a shake flask starting from 1.2 mL of glycerol stocks (30%) stored at −80 °C. The medium composition for the pre-culture was (all in g/kg): carbon source 20 galactose, 2.3 urea, 3.0 KH_2_PO_4_, 0.5 MgSO_4_·7H_2_O, trace element solution 1 g/kg (Stock solution: 15.0 EDTA 2H_2_O, 4.5 ZnSO_4_ 7H_2_O, 1.0 MnCl_2_ 2H_2_O, 0.3 CoCl_2_ 6H_2_O, 0.3 CuSO_4_ 5H_2_O, 0.4 Na_2_MoO_4_ 2H_2_O, 4.5 CaCl_2_ 2H_2_O, 3.0 FeSO_4_ 7H_2_O, 1.0 H_3_BO_3_, 0.1 KI) and 1 g/kg vitamin solution (Stock: 0.05 Biotin, 1.0 Ca-Pantothenate, 1.0 Nicotinic acid, 25.0 Myo-inositol, 1.0 Thiamine chloride, 1.0 Pyridoxol hydrochloride, 0.2 *p*-Aminobenzoic acid). Additionally, 3 mL of chalk solution (0.1 g/g CaCO_3_) were added to a total of 300 g of seed pre-culture medium. The pre-culture was finished after 3 days at 30 °C and 220 rpm reaching a final optical density (600 nm) of about 7. All chemicals were obtained from Sigma-Aldrich, with the exception of biotin, pyridoxol hydrochloride, and pantothenate which were obtained from Merck, thiamine from VWR International BV and EDTA was purchased from Roche.

## Seed fermentation

The seed cultivation was performed in a 7 L bioreactor (Applikon Biotechnology B.V., Delft, The Netherlands) with a maximal working volume of 4 L. The reactor temperature was kept constant at 30 °C; dissolved oxygen tension (DOT) was monitored online using an oxygen probe (Mettler-Toledo, Tiel, The Netherlands). The DO was controlled at a minimum of 20% air saturation via automatic adjustment of the stirring speed. The bioreactor had an overpressure of 0.3 bar, and the aeration rate was 1 vvm (sterile air).

The cultivation was started by transferring 78 mL of the seed pre-culture (OD 7) into the bioreactor containing 2.4 L of fermentation medium at pH 5. The medium contained (all in g/kg): 2 glucose, 1 (NH_4_)_2_SO_4_, 10 KH_2_PO_4_, 5 MgSO_4_·7H_2_O, 8 µL trace element stock solution and 8 µL vitamin stock solution (see pre-culture medium). After depletion of the initial carbon source (detected by a decrease in CO_2_), the feed phase (fedbatch) was started. The feed medium contained 500 g/L glucose and was controlled at an exponential feeding rate of 0.2·e^0.1·t^ (g/kg/h) with a maximum feed rate of 9.2 g/kg/h. After about 48 h, a broth weight of 3.69 kg was reached with a final biomass amount of 53.3 g_CDW_.

## Production cultivation conditions

The biomass from the seed cultivation was diluted to OD 15 – this was achieved by transfer of 285 mL from the seed fermentation, addition of 200 mL of chalk solution (CaCO_3_) and 2.56 kg of the production fermentation medium. The medium contained (in g/kg): 1 urea, 1.5 KH_2_PO_4_, 0.5 MgSO_4_·7H_2_O, 5 iron stock solution (in g/kg: 3 FeSO_4_·7H_2_O, 15 EDTA) and 0.001 biotin. This production fermentation phase was running for more than 90 h, at 30 °C, gassed with a mixture of 1:1 air:CO_2_ at a flow of 0.3 vvm. During the production phase the DO was controlled at 10% air saturation. The cultivation feeding rate was initially set to a constant feeding rate of 9.74 g/h of a 500 g/L glucose solution and was manually decreased at predefined time points (>to prevent glucose accumulation. To keep the pH above 3, chalk solution was added manually at 52 h (100 mL), and at 76 h (100 mL).

### Bioscope labeling experiment

**Fig. 1:**
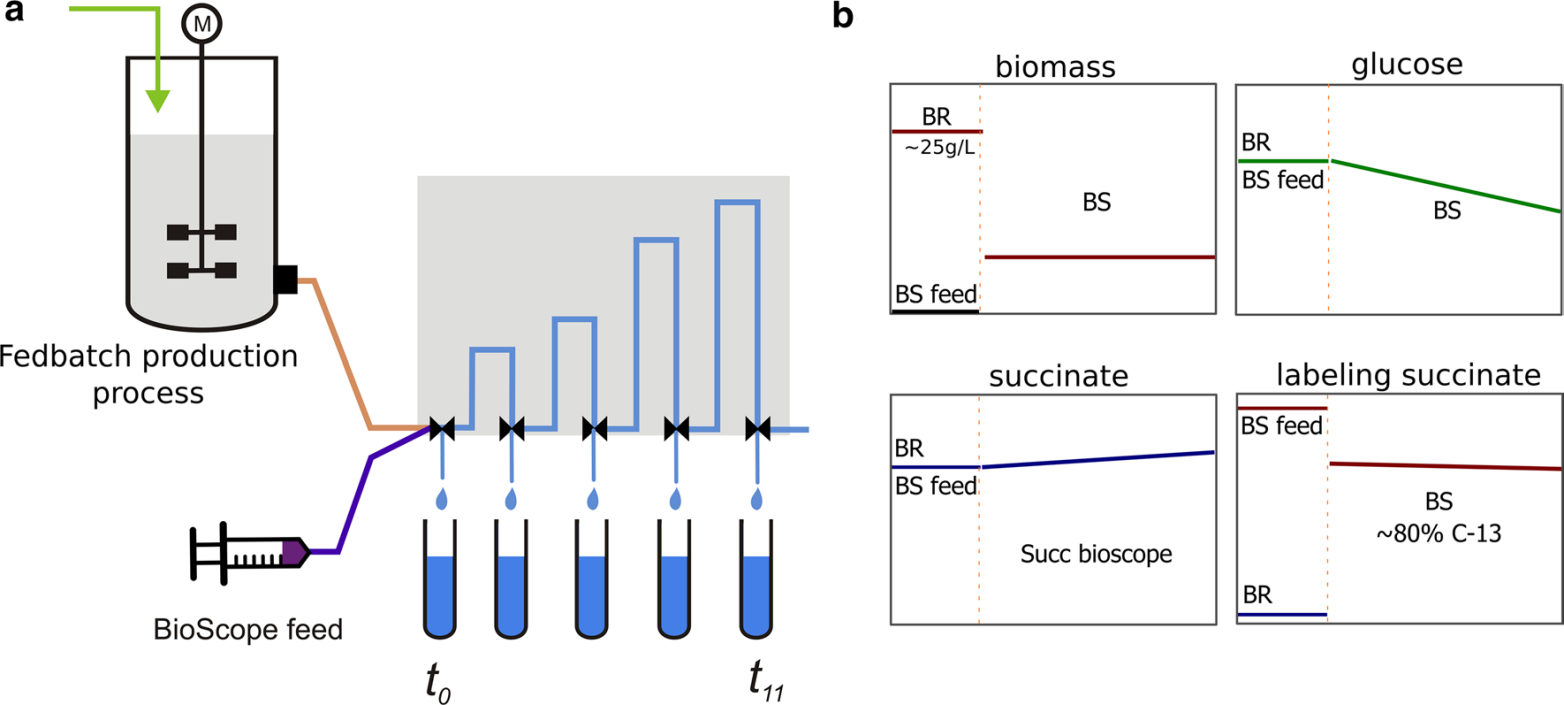
Setup of the bioscope labeling experiment. **a** The bioscope has inflows from the bioreactor (flow 0.14 mL/min) and the BioScope feed (0.56 mL/min). **b** The BioScope feed contains labeled succinic acid matching the concentration of succinic acid of the bioreactor broth. The solution also matches the residual glucose concentration (unlabeled). This setup leads to a dilution of the biomass concentration while the residual concentration of glucose and succinic acid are similar to the BR cultivation. Using labeled succinic acid allows for immediate labeling with about 80% enrichment

Aim of the bioscope labeling experiments was to achieve a fast labeling gradient in the extracellular space without disturbing the metabolic steady-state. The experiments were performed during two phases of the production phase. The BioScope [18], a plug-flow ‘satellite’ reactor, was connected to the fed-batch production cultivation (see Fig. 1). The bioscope reactor inflows were set at the following rates: (1) feeding solution at 0.56 mL/min, (2) inflow from the bioreactor: 0.14 mL/min.

These rates lead to a 5-fold dilution of the bioreactor broth, including the biomass concentration. The concentrations of glucose and succinic acid in the BioScope feed were chosen the same as the extracellular concentrations, to avoid changes in extracellular concentrations after the dilution. Equivalent to the bioreactor a mixture of 1:1 CO_2_ and air was passed through the gas channel of the BioScope. To mimic the flow of 0.5 vvm in the bioreactor a flow rate of 0.086 L/min was used.

### Intracellular and extracellular sampling

Samples for intracellular metabolite quantification were withdrawn using a dedicated rapid sampling device (for bioreactor samples) and quenched in 6 mL −40 °C pure methanol [19]. From this sample 1/3 was further processed—the quenched biomass was poured on a pre-cooled filter and the filter cake was washed with 30 mL methanol cooled at −40 °C [20]. The filter was rapidly transferred into a falcon tube containing 15 mL of 75 °C ethanol and 120 µL of ^13^C cell extract was added. Extraction of the intracellular metabolites was achieved by water bath boiling (3 min at 95 °C) [21].

Samples from the BioScope were collected for about 2 min (~1.4 mL) at each port, weighted and the complete volume was processed by filtration and extraction as in the case of a bioreactor sample.

Extracellular samples from the BioScope were obtained by vacuum filtration [18]. A vial connected to a vacuum pump was equipped with a syringe filter and placed at the bioscope port for about 1.5 min to collect about 1.0 mL of filtrate. The sample was stored at −80 °C until further processing. To determine the concentration in the extracellular space, 100 µL of the filtrate was transferred to a GC vial containing 20 µL of ^13^C cell extract as internal standard and processed comparable to intracellular samples.

### Model setup

A model was developed based on the stoichiometry and atom-transitions listed in Additional file 1: Table S1. The model equations were generated automatically based on model definition files. The labeling of extracellular succinic acid was introduced as initial value for the respective labeling state variables for succinic acid.

Compartmentation was taken into account by the introduction of a cytosolic and a mitochondrial metabolite pool (index _cyt and _mit). The simulation will predict separate enrichment patterns. The concentration of each pool depends on its distribution *f*. The variables f_cyt_Succ, f_cyt_Mal, etc. reflect the (amount) fraction present in the cytosol. All intracellular rates are described as rate per amount of biomass dry weight (g_CDW_) and pools are balanced as amounts per biomass (µmol/g).

The network consists of 31 balanced metabolites, and 39 net- and 24 exchange fluxes (the complete model is documented in Additional file 1, B). Additionally, there are seven parameters for the distribution of TCA cycle intermediates between cytosol and mitochondria. Four fluxes were measured (glucose uptake, and succinate, malate and glycerol production), resulting in 28 fluxes to be estimated. Several (8) of those were excluded from the parameter estimation and set to a fixed value, as these could not be determined from the observed enrichment patterns (and in turn had no impact on the enrichment pattern). Especially, glycolytic exchange fluxes or exchange fluxes in the pentose phosphate pathway were not identifiable as no labeling enrichment was observed within the given observation time. The complete network and a priori fixed fluxes can be found in Additional file 1.

### Simulation and parameter estimation

All simulations and parameter estimations were performed using the simulation software gPROMS 4.0 [22]. The gPROMS model was generated based on the network stoichiometry and known atom transitions using a self-developed model parser.

The parameter estimation was performed using the implemented NLSQ solver. The flux estimation was manually repeated from different starting values and typically converged to comparable minima solutions, especially for fluxes that were estimated with high confidence (see Additional file 1: Tables S2, S3). The confidence intervals were calculated based on linear error propagation (i.e. linearization of the equation system at the measurement timepoints) that is integrated in the gPROMS software package. For the mass isotopomer measurements an error of 3% (absolute) was assumed. Exception is the measurement of Oaa which was less reliable because of the very low concentration of Oaa. Here 30% error were used. This estimated errors not only reflects the MS noise but also the non-ideal plug-flow within the BioScope reactor [23], i.e. the sampling time point is a (narrow) distribution of different time points. The errors of the MS/MS measurements itself are estimated to be between 0.5 and 2% [24]. The standard deviation of the biomass specific extracellular rates was also calculated using linearized error propagation of the black-box model using PWA rate functions. The required standard deviations of the observables (i.e. succininc acid, biomass and other concentrations) was calculated from the repetitive measurements, i.e. the average standard deviation of all observed timepoints.

## Results

### Extracellular metabolic rates

**Fig. 2:**
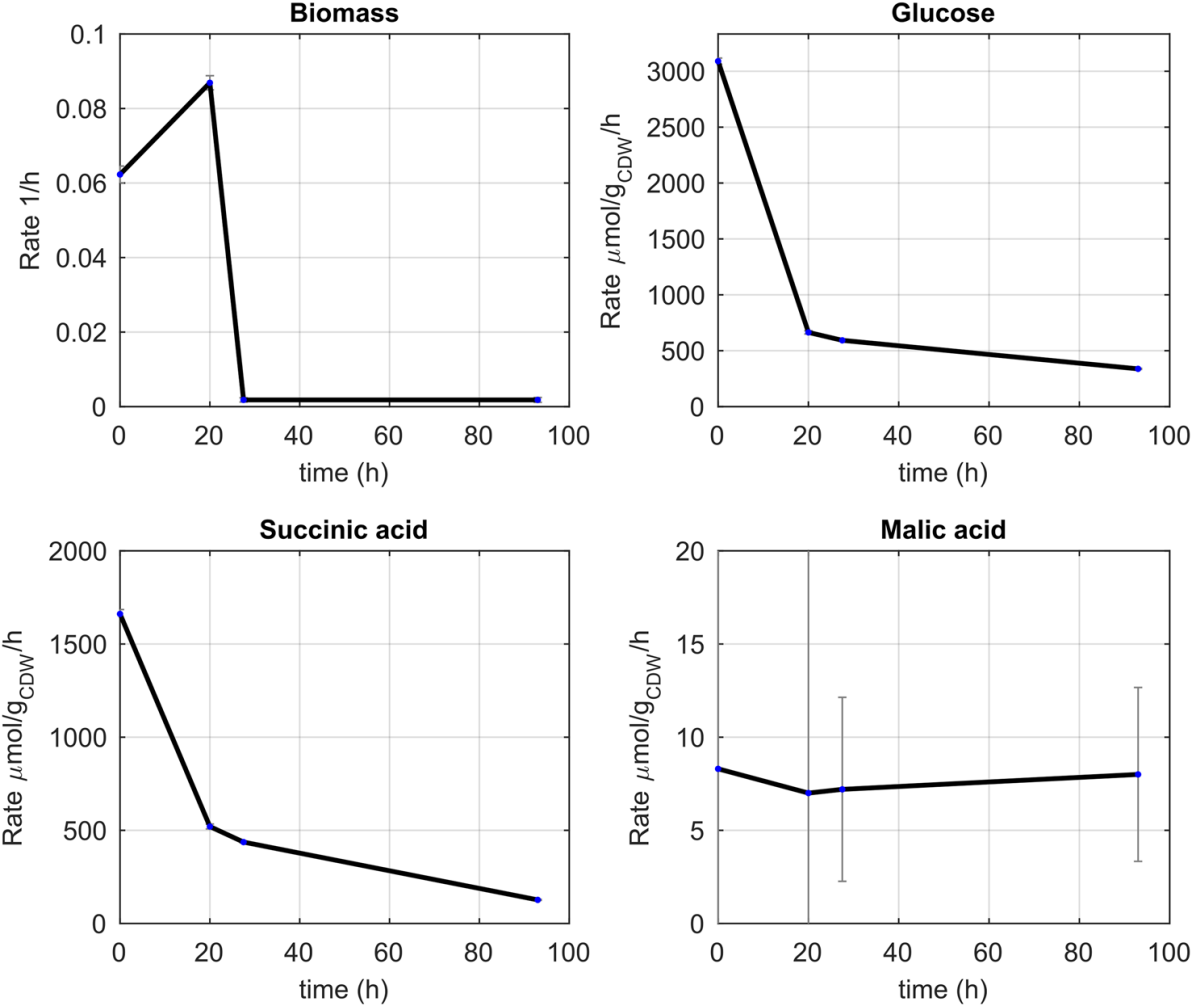
Estimated biomass specific rates over the cultivation time. Table 2 displays the rates at the time of the bioscope experiments. Standard deviations are calculated using linear error propagation (i.e. linearization of the ODE model)

The rates of substrate uptake, succinic acid production and by-product formation were determined from the medium inflow and concentration measurements in time of the bioreactor cultivation using a dynamic flux estimation approach (see Table 2; Additional file 1, A). During the initial phase of the production process (0–24 h), there is biomass growth at an average rate of 0.07 h^−1^ (Fig. 2). During this phase, the glucose uptake rate is high (average of about 1.5 mmol/g/h) and then decreases till the end of the cultivation. Similarly, the succinic acid production rate decreases in time while malic acid is excreted at a constant rate during the process. These rates reproduce the measured concentration profile (Additional file 1: Figure S1). Please note that the residual glucose concentration is the result of feed and uptake rates and reacts very sensitive due to the high biomass concentration and rates. Thus, the deviations observed in Additional file 1: Figure S1 actually reflect small (relative) misfits of the uptake rate.

### Intracellular metabolite concentrations

The intracellular metabolite quantifications show that the levels of three intermediates of the TCA cycle are significantly higher than the others, namely succinate, malate and citrate (Table 4). Compared to wild-type strains at similar glucose uptake rate [25], the concentrations are 100, 50 and 4-fold increased for succinate, malate and citrate, respectively. The concentration of these metabolites seems to increase with increasing extracellular succinate. Conversely intracellular pyruvate as well as α-ketoglutarate decrease with production time and product concentration.

**Fig. 3:**
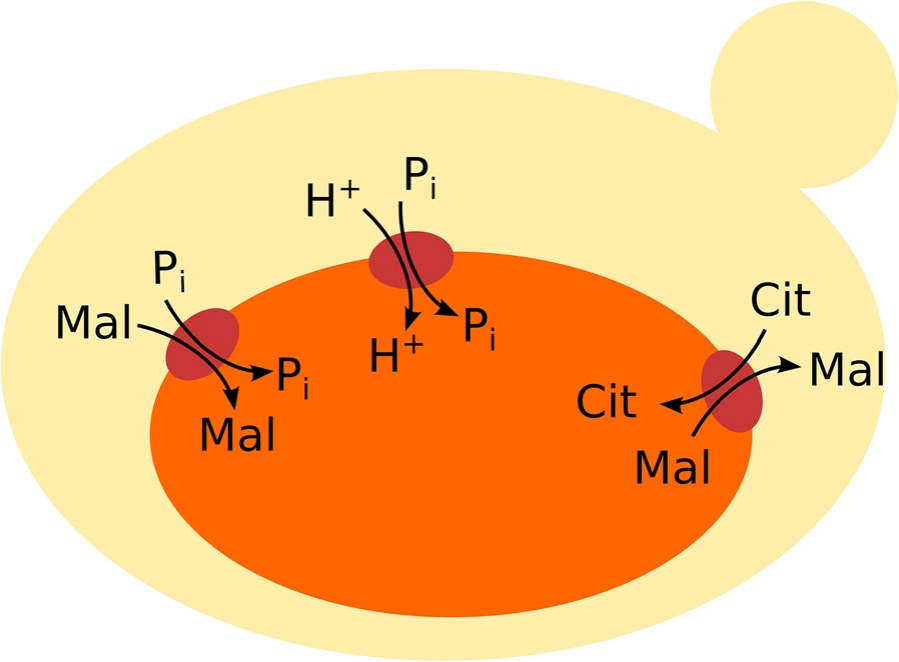
Scheme of intracellular transport reactions of TCA cycle intermediates. A complete list of assumed transport reactions can be found in Table 1, estimated rates are displayed in Figs. 5 and 7

### Identification of product recycling fluxes

Earlier findings suggested that there is a high bidirectional flux of succinic acid over the cellular membrane [26]. This observation was exploited in this study by introducing the ^13^C tracer via succinic acid, labeled on C_1_ and C_4_.Wash-in labeling experiments were performed at two time points of the cultivation, 52 and 73 h, e.g. during the mid-production and late production phase respectively. The intracellular and extracellular metabolite concentrations in the bioreactor were measured before and after the bioscope experiment which took about 1.5 h. At the mid production stage (t = 52 h) an extracellular succinic acid concentration of 490 mmol/L was reached, and 590 mmol/L at t = 73 h. These concentrations were used for the bioscope feed medium. For the estimation of intracellular fluxes, (quasi) steady-state was assumed during the labeling experiment. Please note that the concentration as well as the production flux of succinic acid changed less than 2% during the labeling experiment (1.5 h).

### Compartmentation of the TCA cycle intermediates

The distribution of intracellular TCA cycle intermediates between the cytosol and mitochondria cannot be determined by direct measurements. Citrate (Cit), isocitrate (iCit), α-ketoglutarate (aKG), succinate (Succ), malate (Mal), fumarate (Fum) and oxaloacetate (Oaa) are present in the mitochondria as well as in the cytosol. The distribution can significantly influence the metabolic engineering strategy [27]. Transport studies suggest that there are several transport proteins involved in shuttling metabolites between the cytosol and mitochondria, especially the phosphate/proton symport, Mal/Pi carrier, aKG/Mal carrier and Cit/Mal carrier (Fig. 3, Table 1 and references therein).

**Table 1:** Implemented mitochondrial transporters

Gene	Description	Reaction	Reference
YMR241 W	Citrate and oxoglutarate carrier protein	akg_cyt + cit_mit → akg_mit + cit_cyt	[34]
YBR291C	Citrate transport protein	cit_cyt + mal_mit ⇔ cit_mit + mal_cyt	[35]
YBR291C	Citrate transport protein	cit_cyt + icit_mit ⇔ cit_mit + icit_cyt	[35]
YLR348C	Mitochondrial dicarboxylate carrier	mal _cyt + pi_mit ⇔ mal _mit + pi_cyt	[34]
YKL120 W	Oxaloacetate carrier	h_cyt + oaa_cyt ⇔ h_mit + oaa_mit	[36]
YLR348C	Mitochondrial dicarboxylate carrier	pi_mit + succ_cyt → pi_cyt + succ_mit	[34]
YJR095 W	Mitochondrial succinate-fumarate transporter	fum_mit + succ_cyt → fum_cyt + succ_mit	[36]

The reactions were derived from the genome scale model iMM904 [33] and references found for the respective genes

Assuming that these transporters operate close to equilibrium, the distribution of all TCA cycle intermediates can be calculated (see Additional file 1 for detailed calculations and assumptions). Please note that these calculations are subject to assumptions, i.e. transport equilibrium, subcellular volume distribution and electrochemical potentials. The calculations are used to obtain a theoretical reference for comparison, but were not used in the flux estimation.

### Mid-production phase (t = 52 h)

**Fig. 4:**
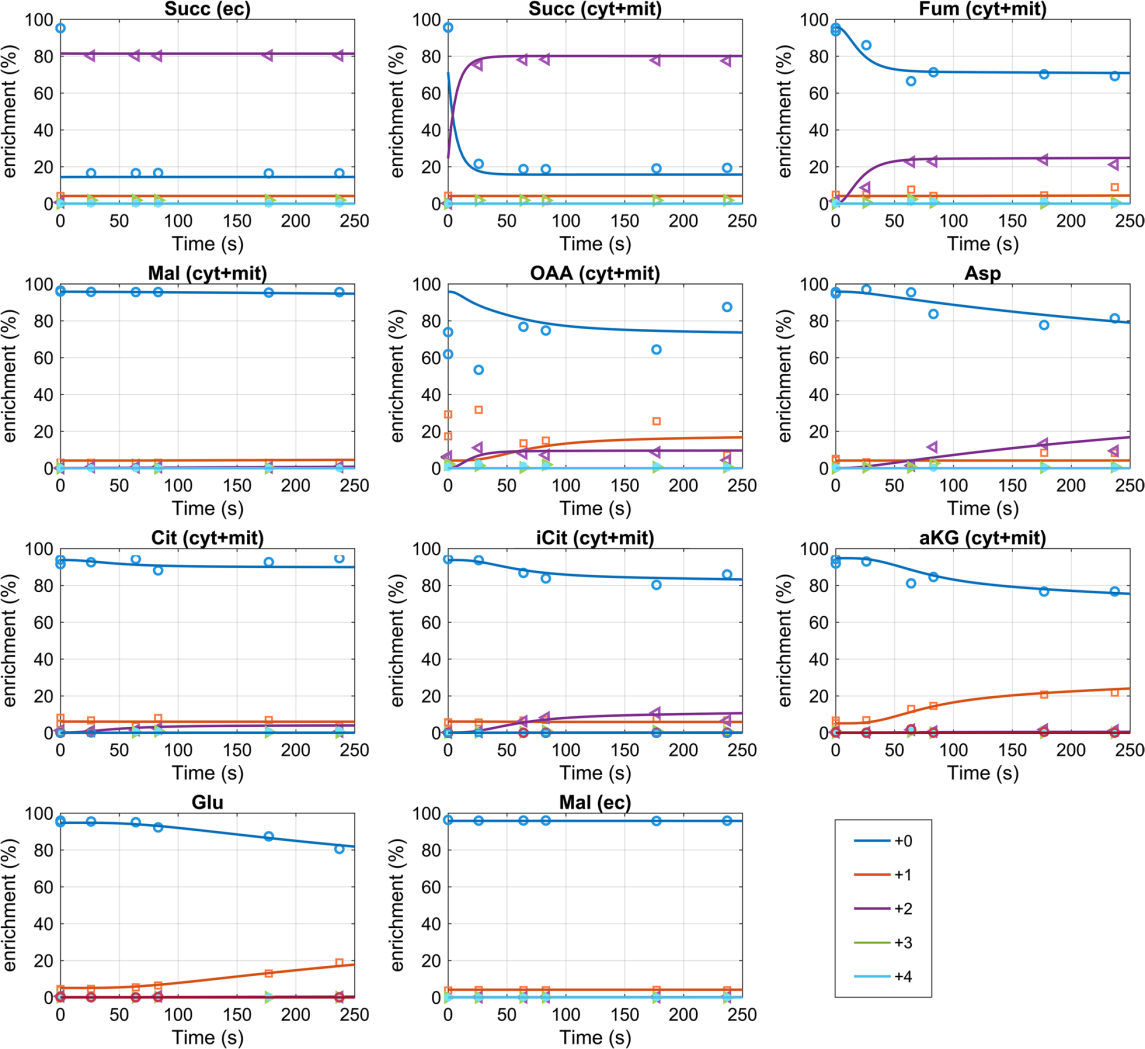
Enrichment measurements and mass isotopomer simulation for t = 52 h. The mass isotopomer simulation represents the sum of the mitochondrial and cytosolic prediction. The measurements for Oaa were less accurate because of the low intracellular concentration and were included in the parameter estimation with a 10× higher standard deviation. The mass isotopomer measurements can be found in Additional file 1: Table S6

The succinic acid labeling nearly reaches the extracellular enrichment at the first sampling time point (t = 18 s), this indicates a very rapid exchange between intra- and extracellular succinic acid pools. A fast increase in ^13^C enrichment was also observed for fumarate, reaching 28% (+2) after about 180 s (Fig. 4). Additionally, enrichment is observed in isocitrate and α-ketoglutarate, reaching 11 and 18%, respectively. Note that for α-ketoglutarate the labeling is observed for the +1 trace, while it is +2 for isocitrate which is in agreement with the decarboxylation. The enrichment in +1 α-ketoglutarate and +2 isocitrate from succinate as labeling source can only be obtained via the oxidative TCA cycle, or a reverse isocitrate lyase, and malate synthase for providing glyoxylate (or alternative enzymes [28]). In both cases, malate is required, with the difference that labeled malate would only be required for the full oxidative cycle. From the experimental measurements, malate reaches a maximal enrichment of about 2% (+2), citrate about 5% (+2). This discrepancy in labelling enrichments of intermediates suggest the compartmentalization of oxidative and reductive TCA cycle activities.

Furthermore, fumarase is a reversible reaction with an equilibrium constant (*K*_*eq*_) of about 4 (for the oxidative direction). To obtain malate from fumarate, the ratio Mal/Fum has to be below 4. The intracellular measurements show values between 150 and 185, for t = 52 h and t = 73 h respectively (Table 2). In an extreme scenario to enable oxidative and reductive directions, all fumarate would be in the mitochondria and (nearly) all malate in the cytosol (scenario 2 in Table 3).

**Table 2:** Biomass specific conversion rates at the time points of the BioScope experiments

Growth	Glucose	Succinic acid	Malic acid	Glycerol
1/h	μmol/g/h	μmol/g/h	μmol/g/h	μmol/g/h
t = 52 h				
0.0018 ± 0.0006	497.4 ± 5.3	320.7 ± 3.5	7.5 ± 4.8	0.9 ± 0.2
t = 73 h				
0.0018 ± 0.0006	415.6 ± 4.7	221.3 ± 3.0	7.8 ± 4.7	1.0 ± 0.2

The standard deviations are calculated from linear error propagation using the blackbox model with piecewise linear rate functions

**Table 3:** Thermodynamic comparison of different scenarios (1) Equilibrium of Mal/Pi transport (2) Derived from the assumption of oxidative fumarase in the mitochondria (Mal/Fum = 3.5)

	Mal/Pi at equilibrium		Mitochondrial fumarase in oxidative direction	
	Conc. ratio (M/C)	Cytosolic amount (%)	Conc. ratio (M/C)	Cytosolic amount
Malate	9.83	50	0.236	98.1%
Succinate	9.59	51		
Fumarate	9.97	50	Inf	0
Citrate	25.51	28.1	0.612	93.9%
Isocitrate	25.41	28.2	0.610	93.9%
aKG	9.96	50.1	0.239	97.6%

The equilibrium calculations depend on assumptions on the electrochemical potential (pmf = −180 mV), the pH difference (ΔpH = 0.5) and volume distribution (cytosol 70%, mitochondria 7%)

**Table 4:** Intracellular metabolite amounts (μmol/g_CDW_)

Time (h)	Succ	Fum	Mal	Cit	iCit	aKG	Pyr	Glx
51.3	71.8	0.42	63.8	25.0	0.80	1.41	0.45	0.18
72.6	94.6	0.50	92.7	35.4	1.76	0.93	0.24	0.13

The measurements were performed just before the bioscope labeling experiment (single measurement at port 0)

In the cytosol malate is synthesized from glucose, which will have a natural enrichment (1.1% ^13^C). In contrast to cytosolic malate, mitochondrial malate will mostly originate from the oxidative TCA cycle, carrying labeling from succinate, but because of the small amount compared to the cytosolic amount, no labeling is observed in the whole cell measurements. In the case of isocitrate, fumarate and α-ketoglutarate there seems to be an accumulation in the mitochondria and therefore ^13^C enrichment can be observed from the whole-cell measurements.

The enrichment in oxaloacetate has been measured as well, however, because of the low concentration in the cell as well as instability during the extraction, the measurements contain significant noise. Nevertheless, a fast enrichment trend can be observed that is further supported by the fast enrichment seen in the associated amino acid aspartate. The labeling enrichment in aspartate (resp. oxaloacetate) also renders a putative role of a reverse glyoxylate shunt, because labeling in oxaloacetate could only be obtained from malate. Summarizing, the labeling observations can only be reproduced by a metabolic network that especially features:

- A fast exchange of succinic acid over the plasma- as well as mitochondrial membrane (here about 15 mmol/g_CDW_/h),
- a differential distribution of TCA cycle intermediates between the cytosol and mitochondria,
- a fast exchange of the intermediate oxaloacetate with aspartate, as well as α-ketoglutarate with glutamate.

Specifically, the parameter estimation suggests that malate, succinate and citrate are predominantly (≥95%) cytosolic (in amount). Oxaloacetate, α-ketoglutarate, fumarate and isocitrate are in the range of 40–65% (Table 5).

**Table 5:** Estimated distribution of TCA cycle acids during the fed-batch process (t = 52 h and t = 73 h)

	% Amount cytosol 52 h (%)	% Amount cytosol 73 h (%)	Conc. ratio M/C 52 h	Conc. ratio M/C 73 h
αKG	40.0	40.0	15.0	15.0
Cit	94.9	96.6	0.5	0.3
FUM	40.0	95.5	15.0	0.5
iCit	62.5	78.2	6.0	2.8
Mal	99.6	99.8	0.0	0.0
Oaa	54.7	89.9	8.3	1.1
Suc	99.8	99.8	0.0	0.0

Please note, the concentration ratio depends on assumptions for the subcellular volumes (here cytosol 70%, mitochondria 7%)

**Fig. 5:**
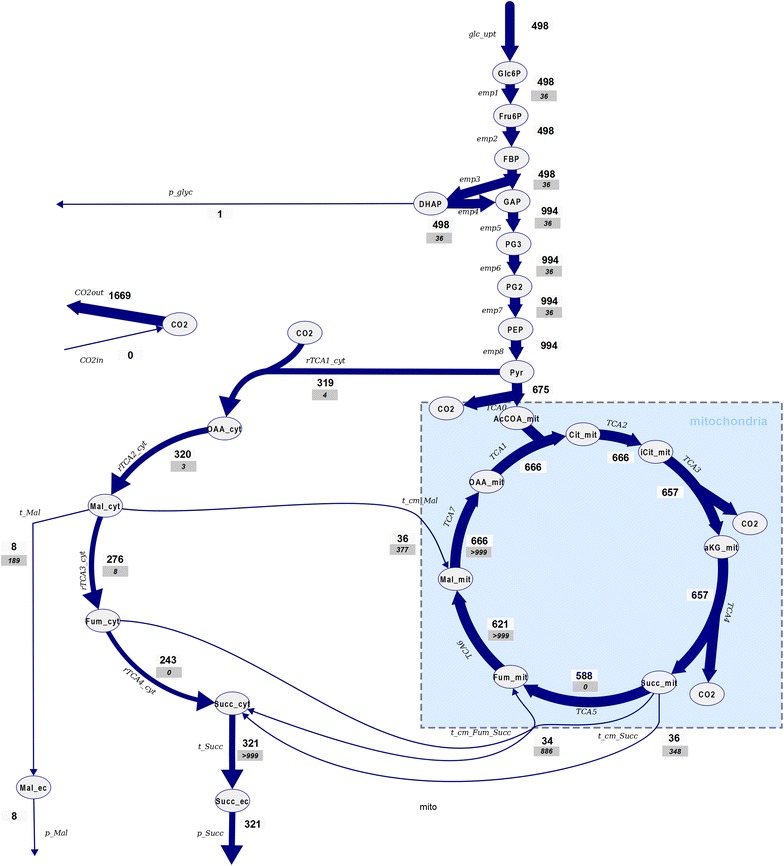
Metabolic flux distribution at t = 52 h. The metabolic fluxes were identified by the addition of labeled succinic acid and parameter estimation. Please note that not all reactions are shown to facilitate the overview. A complete list of reactions can be found in the Additional file 1: Tables S1, S2

At this stage of the cultivation growth is basically absent (due to nitrogen depletion) and the substrate glucose is used for (by-)product formation and maintenance only. At the pyruvate branch point about 1/3 of the carbon enters the reductive route (Fig. 5). The oxidative (mitochondria) and reductive (cytosolic) routes interchange at the level of malate, fumarate as well as succinate. The flux estimation suggests that about 11% of the produced cytosolic malate enters the mitochondria and returns in the form of succinate resp. is catabolized to CO_2_ (this cannot be discriminated). On the one hand, this can be attributed to stoichiometric coupling, i.e. succinate taken from the TCA cycle has to be replenished. On the other hand, the exchange of malate seems reasonable in view of the high intracellular (cytosolic) concentration (Tables 4, 5). The import of malate into the mitochondria leads to a lower flux of malate to fumarate in the cytosol. This finding together with the surprisingly high malate concentration, as well as observing malate as by-product suggest a putative limitation of fumarase in the reductive direction. Although, there is a high thermodynamic driving force, not all malate arriving from oxaloacetate can be converted to fumarate, but is diverted to the TCA cycle and also the extracellular space.

**Fig. 6:**
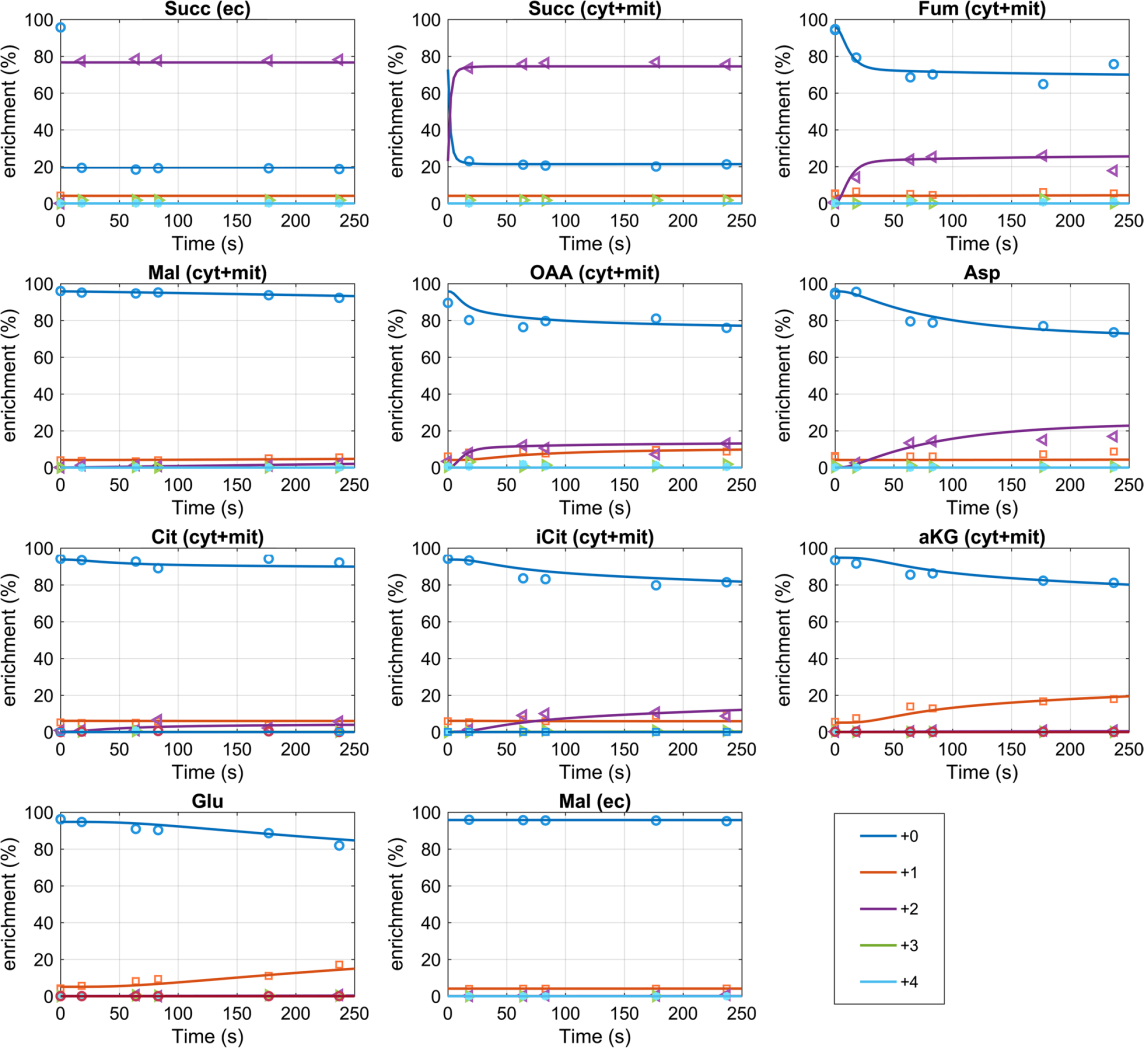
Simulated and measured mass isotopomers for t = 73 h. From the simulation the sum of mass isotopomers from mitochondria and cytosol are calculated, representing the predicted whole-cell measurements. The mass isotopomer measurements can be found in Additional file 1: Table S7

**Fig. 7:**
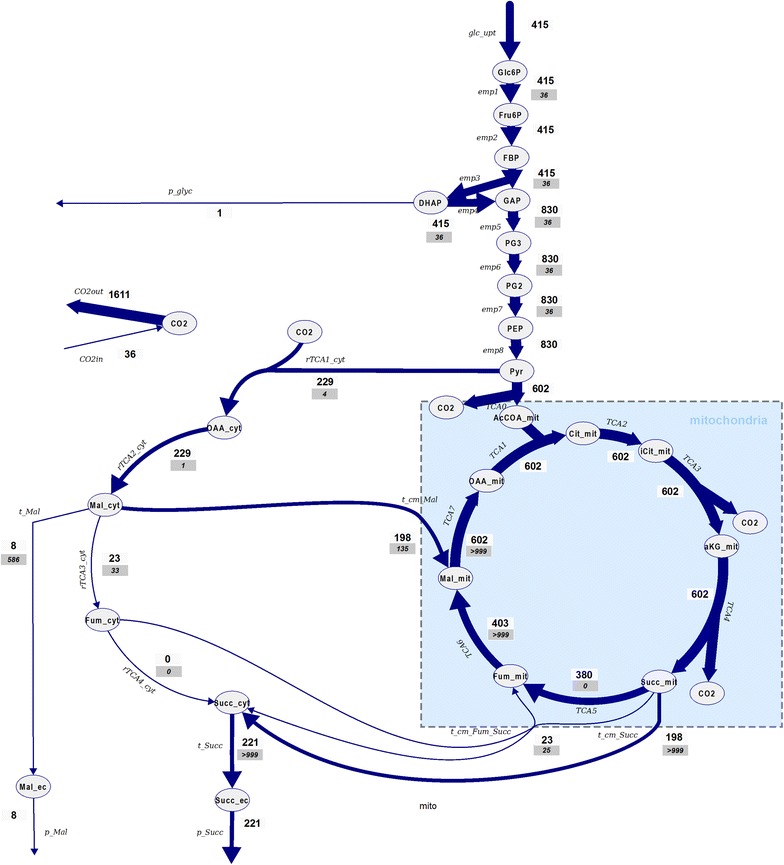
Estimated flux distribution at t = 73 h. All flux values are given in µmol/g/h. Please note that not all reactions are shown to facilitate the overview. A complete list of reactions can be found in the Additional file 1: Tables S1, S3

From the model estimation, the labeling originating from the extracellular labeled succinic acid leads to a high labeling in cytosolic succinate, that interchanges with the mitochondrial pool using the Suc/Pi carrier but also Suc/Fum transporter. The predicted labeling of mitochondrial succinate is 50%. For the downstream metabolites Fum, Mal and Oaa lower enrichments are predicted because of the inflow of unlabeled metabolites from the cytosol, especially the exchange with Fum and Mal.

Unfortunately, but also expected, the parameter estimation has large confidence intervals (Additional file 1: Table S2) and there is significant correlation between transport (exchange) rates and the metabolite distribution variables. To further analyze if the results are consistent, a thermodynamic analysis was performed using the estimated metabolite distribution, compartment volume distribution and previously reported estimates of cofactor ratios (Additional file 1: Table S4). It can be seen that for most reactions the flux direction estimated from the ^13^C model is supported, nevertheless, there is an inconsistency for mitochondrial succinate dehydrogenase (the estimated Fum/Suc ratio is too high). The transport reaction directions are consistent with the flux estimation, except the direction of the Fum/Suc shuttle—for Suc and Fum the largest standard deviations were observed.

Despite the uncertainty and the observed inconsistencies, it has to be noted that the observed labeling requires mechanisms to enable oxidative and reductive TCA cycle activity together with exchange reactions. The available measurements currently only allow an estimation with inaccuracies.

### Late production phase (t = 73 h)

In the late production phase the glucose consumption rate is reduced and the rate and yield of succinic acid decreased. Compared to the mid-production phase, a lower fraction of pyruvate is entering the reductive, cytosolic product pathway. About 68% of the available pyruvate enters the mitochondria and is catabolized in the oxidative TCA cycle. There is some exchange of succinate and fumarate between the cytosol and the mitochondria, i.e. some cytosolic Fum is entering the mitochondria and succinate is excreted to the cytosol, maybe because of a limitation in mitochondrial fumarate reductase flux (*Q/K*_*eq*_ > 1, Additional file 1: Table S5). Additionally, there is a high bidirectional rate for the Suc/Pi carrier, but low net flux. This constellation leads to a lower cytosolic activity of fumarate reductase compared to the previous reaction steps, i.e. fumarase.

Comparing the estimated distribution of acids to the previous phase, the general trend is comparable, but the cytosolic amount of Fum and Oaa is higher (Table 5). There is no observable enrichment in malate, and also citrate only reached a lower enrichment of 2% for the +2 mass. In contrast, α-ketoglutarate +1 reaches 21% after 237 s (Fig. 6) indicating a pool mostly participating in the oxidative TCA cycle (estimated 15 times higher concentration in the mitochondria compared to the cytosol). Isocitrate is reaching 10% enrichment on the +2 mass trace, similar to the mid-production phase.

The flux estimation shows a significant change in metabolic flux distribution, i.e. 86% of the synthesized cytosolic malate now enters the mitochondria. During this phase, the export of malate to the extracellular space is at a comparable rate (8 μmol/g_*CDW*_/h), in relative amounts about 3.4% which is a slight increase compared to the earlier phase (Fig. 7). The intracellular malate concentration at this stage is about 92 µmol/g_CDW_, again suggesting a limiting step in the reductive pathway.

## Conclusions

The metabolic flux analysis of high-producing succinic acid yeast revealed a high recycling of the acid product over the cell membrane (about 15 mmol/g_CDW_/h) as well as exchange between the cytosol and mitochondria. Extracellular, labeled succinic acid was imported and labeled carbon atoms were found back in intermediates of the TCA cycle. Thermodynamic considerations, especially with respect to the required oxidative direction of fumarase in the mitochondria and reductive direction in the cytosol, suggests that malate is mostly present in the cytosol. The high intracellular concentration leads to excretion of malate to the extracellular space but also transport of malate into the mitochondria. Because there is correlation between the cytosolic/mitochondrial malate ratio and the exchange flux, the flux estimation has a comparably high standard deviation. Nevertheless, the labeling measurements require an exchange of malate between the cytosol and mitochondria.

The kinetics of fumarase have been studied in vitro and inhibition by inorganic phosphate has been described [29]. Recent measurements in wild-type strains suggest a cytosolic phosphate concentration of 20 mM, which is the range of the *K*_*I*_ for phosphate inhibition [29]. Unfortunately, no phosphate measurements were available for the strain used here.

For the current work, several assumptions were derived from previous observations or theoretical considerations, rather than direct measurements. While these assumptions influence the concentration ratios and thermodynamic interpretation, the flux and amount ratio estimated from the labeling measurements is not influenced by these assumptions. During the parameter optimization a coefficient representing the amount (units µmol/g, not the compartment specific concentration) ratio was used. Furthermore, the intracellular fluxes were normalized to whole cell amounts (unit µmol/g/h, not compartment specific volumes).

Compartment specific measurements would facilitate and improve the identification of fluxes and ratios. Similar to previous developed sensor reactions for (cytosolic) concentration measurements [30–32], sensor reactions mirroring the labeling enrichment could be introduced and used to obtain mitochondrial or cytosolic labeling enrichments. Further, physiological measurements, especially regarding the intracellular volume distribution under the observed conditions could reduce the amount of assumptions and increase the accuracy for data interpretation like thermodynamics. The distribution of the acids is also influenced by the electrochemical potential and pH difference which have not been measured under the cultivation conditions used here.

On the other hand, the labeling approach can overcome the limitation of direct compartment specific measurements to a certain extend and revealed intracellular product recycling. The measurements clearly show that compartmentation can lead to a condition of bidirectional fluxes, including the catabolism of the desired product. The labeling approach can easily be applied to other production processes and used to identify putative product recycling activity especially for other products derived from the TCA cycle or lactic acid production. Performing the labeling experiment in a secondary reactor like the bioscope furthermore reduces the amount of labeled material required and allows for a series of experiments with different substrate(s) labeling(s). The dilution approach allows to reach quasi steady-state conditions while the labeling state is switched, allowing to identify fast intracellular fluxes.

## Additional file

**Additional file 1.** Supplementary information.
