# Supplementary material for: Intracellular product recycling in high succinic acid producing yeast at low pH
Source: Microb Cell Fact. 2017 May 23;16:90. doi: 10.1186/s12934-017-0702-0 (PMC5442661; doi:10.1186/s12934-017-0702-0)
Supplement: Supplementary file 1 — Additional file 1. Supplementary information. [file 12934_2017_702_MOESM1_ESM.docx]

# Supplementary Material

## Estimation of extracellular rates using a black box model

The metabolic rates change during the fed-batch process. To obtain an estimation of the extracellular rates, a black box model was used, balancing biomass, succinic and malic acid, as well as glucose and glycerol. The biomass specific metabolic rates in time are approximated by piece-wise affine functions (PWA):

$$\mathbf{v}(t)=\left\{\begin{matrix} \mathbf{v}_{0}+(\mathbf{v}_{1}-\mathbf{v}_{0})/t_{1} t & t\leq t_{1} \\ \mathbf{v}_{1}+(\mathbf{v}_{2}-\mathbf{v}_{1})/(t_{2}-t_{1}) ( t-t_{1}) & t_{1}<t\leq t_{2} \\ \mathbf{v}_{2}+(\mathbf{v}_{3}-\mathbf{v}_{2})/(t_{3}-t_{2}) ( t-t_{2}) & t_{2}<t\leq t_{3} \end{matrix}\right.$$

The vector of specific rates contains the biomass growth rate (µ), glucose uptake (*q_S_*), succinic acid production (*q_P_*), as well as *q_Glyc_* (glycerol) and *q_Mal_* (malic acid). The amounts of the balanced compounds are calculated by the respective balances. During the fed-batch experiments, the bioscope was connected at three timepoints, leading to a broth effluent (*F_bios_*) which has to be taken into account:

$$\begin{matrix} \frac{d c_{X}V}{dt}=\mu c_{X} V-c_{X}F_{bios} \\ \frac{d c_{Glc}V}{dt}=F_{in}c_{Glc,in}-q_{Glc} c_{X} V-c_{X}F_{bios} \\ \frac{d c_{Succ}V}{dt}=q_{Succ} c_{X} V-c_{Succ}F_{bios} \\ \frac{d c_{Mal}V}{dt}=q_{Mal} c_{X} V-c_{Mal}F_{bios} \\ \frac{d c_{Glyc}V}{dt}=q_{Glyc} c_{X} V-c_{Glyc}F_{bios} \\ \frac{d V}{dt}=F_{in}-F_{bios} \end{matrix}$$

Parameter estimation, using the particle swarm toolbox PSwarm (version 1.5) [37] was applied to obtain the metabolic rates at the PWA breakpoints (t=0, 21, 27.5, 93h).


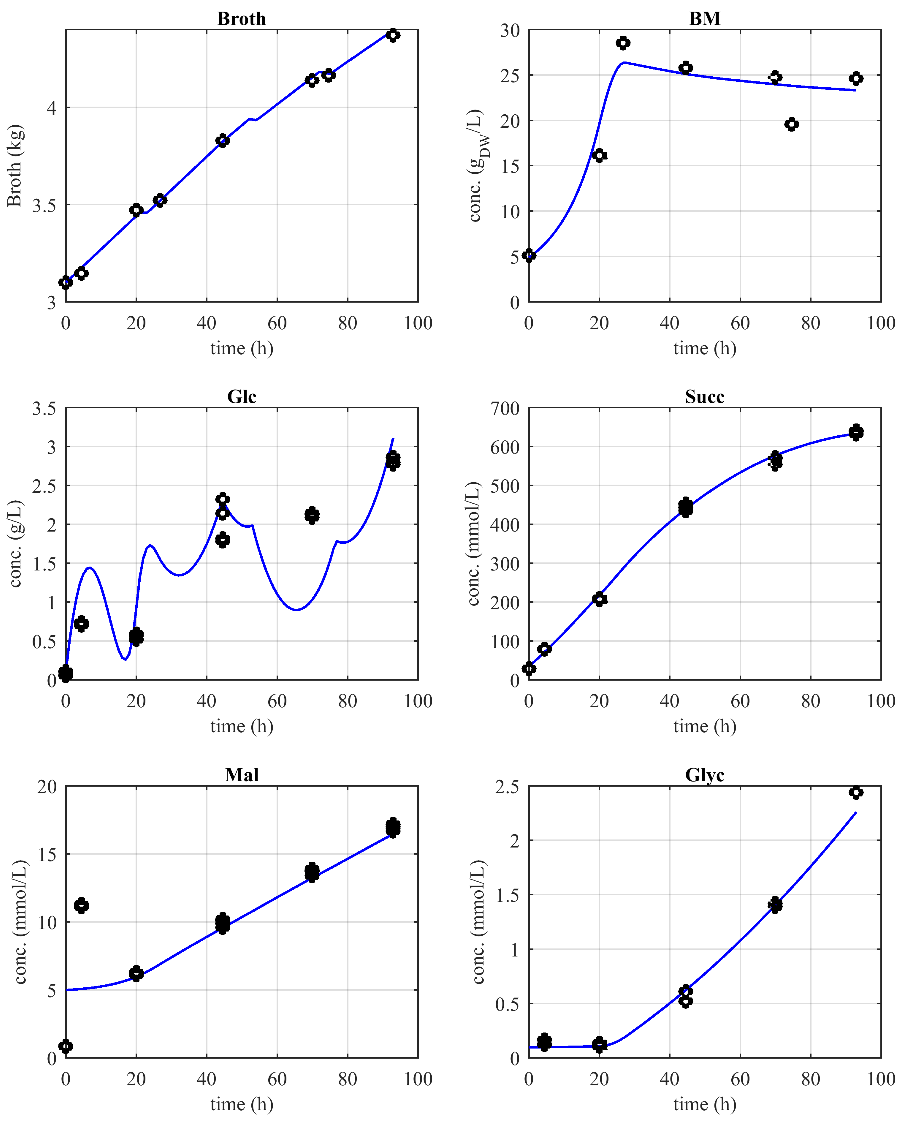


Figure S1: Extracellular metabolite measurements and approximation using a PWA modelling approach for rate estimation.

## B. Metabolic reactions and atom transition network

**Table S1:** Metabolic reactions used for simulation and flux estimation. ‘>’ indicates an irreversible reaction, ‘<>’ indicates bidirectional flux. Atom transitions are displayed in the notation by [38].

| glc_upt | Glc_ec | > | Glc6P |  |  |  |  |
| --- | --- | --- | --- | --- | --- | --- | --- |
|  | #abcdef | > | #abcdef |  |  |  |  |
| t_Succ | Succ_ec | <> | Succ_cyt |  |  |  |  |
|  | #ABCD | > | #ABCD |  |  |  |  |
| p_Succ | Succ_ec | > | Succ_sink |  |  |  |  |
|  | #ABCD | > | #ABCD |  |  |  |  |
| t_Mal | Mal_ec | <> | Mal_cyt |  |  |  |  |
|  | #ABCD | > | #ABCD |  |  |  |  |
| p_Mal | Mal_ec | > | Mal_sink |  |  |  |  |
|  | #ABCD | > | #ABCD |  |  |  |  |
| emp1 | Glc6P | <> | Fru6P |  |  |  |  |
|  | #abcdef | > | #abcdef |  |  |  |  |
| emp2 | Fru6P | > | FBP |  |  |  |  |
|  | #abcdef | > | #abcdef |  |  |  |  |
| emp3 | FBP | <> | DHAP | + | GAP |  |  |
|  | #abcdef | > | #cba | + | #def |  |  |
| emp4 | DHAP | <> | GAP |  |  |  |  |
|  | #cba | > | #abc |  |  |  |  |
| emp5 | GAP | <> | PG3 |  |  |  |  |
|  | #abc | > | #abc |  |  |  |  |
| emp6 | PG3 | <> | PG2 |  |  |  |  |
|  | #abc | > | #abc |  |  |  |  |
| emp7 | PG2 | <> | PEP |  |  |  |  |
|  | #abc | > | #abc |  |  |  |  |
| emp8 | PEP | > | Pyr |  |  |  |  |
|  | #abc | > | #abc |  |  |  |  |
| p_Glyc | GAP | > | Glyc_ec |  |  |  |  |
|  | #abc | > | #abc |  |  |  |  |
| rTCA1_cyt | Pyr | + | CO2 | <> | OAA_cyt |  |  |
|  | #ABC |  | #D | > | #ABCD |  |  |
| rTCA2_cyt | OAA_cyt | <> | Mal_cyt |  |  |  |  |
|  | #ABCD | > | #ABCD |  |  |  |  |
| rTCA3_cyt | Mal_cyt | <> | Fum_cyt |  |  |  |  |
|  | #ABCD | > | #ABCD |  |  |  |  |
| rTCA4_cyt | Fum_cyt | + | Fum_cyt | <> | Succ_cyt | + | Succ_cyt |
|  | #ABCD | + | #abcd | > | #ABCD | + | #dcba |
| t_cm_Succ | Succ_cyt | <> | Succ_mit |  |  |  |  |
|  | #ABCD | > | #ABCD |  |  |  |  |
| t_cm_AcCoA | AcCOA_mit | <> | AcCOA_cyt | |  |  |  |
|  | #ab | > | #ab |  |  |  |  |
| gs1 | iCit_cyt | > | Glx_cyt | + | Succ_cyt |  |  |
|  | #ABCDEF | > | #AB | + | #CDEF |  |  |
| gs2 | AcCOA_cyt | + | Glx_cyt | > | Mal_cyt |  |  |
|  | #AB | + | #ab | > | #abAB |  |  |
| TCA0 | Pyr | > | AcCOA_mit | + | CO2 |  |  |
|  | #abc | > | #bc | + | #a |  |  |
| TCA1 | AcCOA_mit | + | OAA_mit | > | Cit_mit |  |  |
|  | #AB | + | #abcd | > | #dcbaBA |  |  |
| TCA2 | Cit_mit | > | iCit_mit |  |  |  |  |
|  | #ABCDEF | > | #ABCDEF |  |  |  |  |
| TCA3 | iCit_mit | > | aKG_mit | + | CO2 |  |  |
|  | #ABCDEF | > | #ABCEF | + | #D |  |  |
| TCA4 | aKG_mit | > | Succ_mit | + | CO2 |  |  |
|  | #ABCDE | > | #BCDE | + | #A |  |  |
| TCA5 | Succ_mit | + | Succ_mit | <> | Fum_mit | + | Fum_mit |
|  | #ABCD | + | #abcd | > | #ABCD | + | #dcba |
| TCA6 | Fum_mit | <> | Mal_mit |  |  |  |  |
|  | #ABCD | > | #ABCD |  |  |  |  |
| TCA7 | Mal_mit | <> | OAA_mit |  |  |  |  |
|  | #ABCD | > | #ABCD |  |  |  |  |
| t_cm_Fum_Succ | Fum_mit | + | Succ_cyt | <> | Fum_cyt | + | Succ_mit |
|  | #ABCD | + | #abcd | > | #ABCD | + | #abcd |
| t_cm_Mal | Mal_mit | <> | Mal_cyt |  |  |  |  |
|  | #ABCD | > | #ABCD |  |  |  |  |
| t_cm_Cit_Mal | Cit_mit | + | Mal_cyt | <> | Cit_cyt | + | Mal_mit |
|  | #ABCDEF | + | #abcd | > | #ABCDEF | + | #abcd |
| t_cm_iCit_Cit | iCit_mit | + | Cit_cyt | <> | iCit_cyt | + | Cit_mit |
|  | #ABCDEF | + | #abcdef | > | #ABCDEF | + | #abcdef |
| t_cm_aKG_Cit | aKG_mit | + | Cit_cyt | <> | aKG_cyt | + | Cit_mit |
|  | #ABCDE | + | #abcdef | > | #ABCDE | + | #abcdef |
| CO2in | CO2_ec | > | CO2 |  |  |  |  |
|  | #A | > | #A |  |  |  |  |
| CO2out | CO2 | > | CO2_sink |  |  |  |  |
|  | #A | > | #A |  |  |  |  |
| bAsp | OAA | <> | Asp |  |  |  |  |
|  | #abcd | > | #abcd |  |  |  |  |
| bGlu | AKG | <> | Glu |  |  |  |  |
|  | #abcde | > | #abcde |  |  |  |  |

Table S2: Overview for flux estimation including standard deviations based on linear error propagation at t=52h. Fluxes that could not be identified, or reached the boundary during flux estimation were fixed (right table). All values are in µmol/g_CDW_/h.

| Flux | Value | Flux | Value |
| --- | --- | --- | --- |
| bAsp_net | 0.00 ± 0.00 | t_cm_Mal_net | -35.88 ± 1483008 |
| bAsp_xch | 0.05 ± 0.02 | t_cm_Mal_xch | 0.09 ± 49.00 |
| bGlu_net | 0.00 ± 0.00 | t_cm_Succ_net | -35.88 ± 1483008 |
| bGlu_xch | 0.17 ± 0.09 | t_cm_Succ_xch | 0.09 ± 1500 |
| CO2in_net | 0.00 ± 7341 | t_Mal_net | -7.50 ± 4.80 |
| CO2out_net | 1668.90 ± 7341 | t_Mal_xch | 0.05 ± 2.90 |
| emp1_net | 497.40 ± 5.30 | t_Succ_net | -320.70 ± 3.50 |
| emp2_net | 497.40 ± 5.30 | t_Succ_xch | 0.95 ± 5.70 |
| emp3_net | 497.40 ± 5.30 | TCA0_net | 674.46 ± 25.90 |
| emp4_net | 497.40 ± 5.30 | TCA1_net | 665.70 ± 12.15 |
| emp5_net | 993.90 ± 10.60 | TCA2_net | 665.70 ± 12.15 |
| emp6_net | 993.90 ± 10.60 | TCA3_net | 656.94 ± 25.90 |
| emp7_net | 993.90 ± 10.60 | TCA4_net | 656.94 ± 25.90 |
| emp8_net | 993.90 ± 10.60 | TCA5_net | 587.30 ± 25455 |
| glc_upt_net | 497.40 ± 5.30 | TCA5_xch | 0.00 ± 3.60 |
| gs1_net | 8.76 ± 22.88 | TCA6_net | 621.06 ± 1483007 |
| gs2_net | 8.76 ± 22.88 | TCA6_xch | 0.45 ± 290 |
| p_Glyc_net | 0.90 ± 0.20 | TCA7_net | 665.70 ± 12.15 |
| p_Mal_net | 7.50 ± 4.80 | TCA7_xch | 0.42 ± 220 |
| p_Succ_net | 320.70 ± 3.50 |  |  |
| rTCA1_cyt_net | 319.44 ± 23.64 | fixed |
| rTCA2_cyt_net | 319.44 ± 23.64 | **Flux** | **Value** |
| rTCA2_cyt_xch | 0.00 ± 0.43 | emp1_xch | 0.01 |
| rTCA3_cyt_net | 276.06 ± 1483007 | emp3_xch | 0.01 |
| rTCA3_cyt_xch | 0.00 ± 71.00 | emp4_xch | 0.01 |
| rTCA4_cyt_net | 121.15 ± 25456 | emp5_xch | 0.01 |
| rTCA4_cyt_xch | 0.00 ± 2.40 | emp6_xch | 0.01 |
| t_cm_AcCoA_net | 8.76 ± 22.88 | emp7_xch | 0.01 |
| t_cm_aKG_Cit_net | 0.00 ± 0.00 | rTCA1_cyt_xch | 0 |
| t_cm_Cit_Mal_net | 8.76 ± 22.88 | t_cm_AcCoA_xch | 0 |
| t_cm_Fum_Succ_net | -33.76 ± 1451206 | t_cm_aKG_Cit_xch | 0 |
| t_cm_Fum_Succ_xch | 0.20 ± 1200 | t_cm_Cit_Mal_xch | 0 |
| t_cm_iCit_Cit_net | 8.76 ± 22.88 | t_cm_iCit_Cit_xch | 0 |

Table S3: Overview for flux estimation including standard deviations based on linear error propagation at t=73h. Fluxes that could not be identified, or reached the boundary during flux estimation were fixed (right table). All values are in µmol/g_CDW_/h.

| Flux | Value | Flux | Value |
| --- | --- | --- | --- |
| bAsp_net | 0.00 ± 0.00 | TCA0_net | 601.10 ± 10.93 |
| bAsp_xch | 0.00 ± 0.00 | TCA1_net | 601.10 ± 10.93 |
| bGlu_net | 0.00 ± 0.00 | TCA2_net | 601.10 ± 10.93 |
| bGlu_xch | 0.14 ± 0.08 | TCA3_net | 601.10 ± 10.93 |
| CO2out_net | 1610.20 ± 35.96 | TCA4_net | 601.10 ± 10.93 |
| emp1_net | 415.60 ± 4.70 | TCA5_net | 190.05 ± 50.37 |
| emp2_net | 415.60 ± 4.70 | TCA6_net | 385.11 ± 163.24 |
| emp3_net | 415.60 ± 4.70 | TCA7_net | 601.10 ± 10.93 |
| emp4_net | 415.60 ± 4.70 |  |  |
| emp5_net | 830.20 ± 9.40 | **fixed** | |
| emp6_net | 830.20 ± 9.40 | **Flux** | **Value** |
| emp7_net | 830.20 ± 9.40 | CO2in_net | 36 |
| emp8_net | 830.20 ± 9.40 | gs1_net | 0 |
| glc_upt_net | 415.60 ± 4.70 | rTCA1_cyt_xch | 0 |
| gs2_net | 0.00 ± 0.00 | rTCA2_cyt_xch | 0 |
| p_Glyc_net | 1.00 ± 0.20 | rTCA3_cyt_xch | 0.01 |
| p_Mal_net | 7.80 ± 4.70 | rTCA4_cyt_xch | 0 |
| p_Succ_net | 221.30 ± 3.00 | t_cm_aKG_Cit_xch | 0 |
| rTCA1_cyt_net | 229.10 ± 5.58 | t_cm_Cit_Mal_xch | 0 |
| rTCA2_cyt_net | 229.10 ± 5.58 | t_cm_Mal_xch | 0.03 |
| rTCA3_cyt_net | 5.31 ± 162.91 | t_cm_Succ_xch | 0.45 |
| rTCA4_cyt_net | 0.15 ± 50.10 | t_Mal_xch | 0.14 |
| t_cm_AcCoA_net | 0.00 ± 0.00 | TCA5_xch | 0 |
| t_cm_aKG_Cit_net | 0.00 ± 0.00 | TCA6_xch | 0.47 |
| t_cm_Cit_Mal_net | 0.00 ± 0.00 | TCA7_xch | 0.52 |
| t_cm_Fum_Succ_net | -5.01 ± 180.83 | emp1_xch | 0.01 |
| t_cm_Fum_Succ_xch | 0.01 ± 0.06 | emp3_xch | 0.01 |
| t_cm_iCit_Cit_net | 0.00 ± 0.00 | emp4_xch | 0.01 |
| t_cm_Mal_net | -215.99 ± 162.88 | emp5_xch | 0.01 |
| t_cm_Succ_net | -215.99 ± 162.88 | emp6_xch | 0.01 |
| t_Mal_net | -7.80 ± 4.70 | emp7_xch | 0.01 |
| t_Succ_net | -221.30 ± 3.00 | t_cm_AcCoA_xch | 0 |
| t_Succ_xch | 0.94 ± 0.21 | t_cm_iCit_Cit_xch | 0 |

## Transport equilibrium calculation details

The following assumptions were used for these calculations: pH cytosol (pH_C_ = 6.8), pH mitochondria (pH_M_ = 7.3), electrochemical potential difference (mitochondria vs. cytosol) $\psi_{M}=-180\text{ }mV$ [39], the volume of the mitochondria 7% of the cellular volume [40], the volume of the cytosol ~70% of the total cellular volume.

Especially the exchange of inorganic phosphate has a significant influence. The reaction reads:

Please note that dihydrogen phosphate ion is the only transported species. Taking into account the differences in pH between cytosol and mitochondria and the membrane potential, the following distributions are obtained:

$$\frac{\left(H_{2}PO_{4}^{-}\right) _{M}}{\left(H_{2}PO_{4}^{-}\right)_{C}}=10^{pH_{M}-pH_{C}}=3.16 \frac{\left(HPO_{4}^{2-}\right) _{M}}{\left(HPO_{4}^{2-}\right)_{C}}=10^{2pH_{M}-2pH_{C}}$$

It should be realized that there could be further processes involved in the total phosphate equilibrium, like the formation of a magnesium phosphate complex – nevertheless for the current study the equilibrium of the hydrogen phosphate ion is most relevant for the other carriers, like the Malate/Pi carrier:

The measured malate concentration is, however, the sum of all species. Replacing Mal^2-^ with the respective fraction of the total concentration at the mitochondrial / cytosolic pH the expected ratio at equilibrium is obtained:

The same approach can be applied for the other Pi dependent shuttles (see Table 4 for the ratios).

For the other shuttles, e.g. the citrate/malate carrier the following stoichiometry is reported:

Under the assumption of equilibrium, the following distribution is obtained:

$$\frac{\left(Cit^{x}\right) _{M}}{\left(Cit^{x}\right)_{C}}= \frac{ 10^{3pH_{M}}+10^{2pH_{M}+pKa3}+10^{pH_{M}+pKa2+pKa3}+10^{pKa1+pKa2+pKa3}}{ 10^{3pH_{C}}+10^{2pH_{C}+pKa3}+10^{pH_{C}+pKa2+pKa3}+10^{pKa1+pKa2+pKa3}} =25.5$$

Similar calculations were performed for the other carriers (see ). Assuming that this equilibrium is reached *in-vivo* and the volume distribution reported in literature is valid for the succinic acid producing mutant under the conditions applied, the measured amounts will reflect about 42% mitochondrial molecules (Mal, Succ, Fum) (about 65% for Citrate). If this equilibrium is indeed reached is questionable, nevertheless the values give first expectations and help in the interpretation of transport activities, especially when placed in the context of other equilibria.

A first observation is that the calculated distribution of malate is in conflict with the resulting malate/fumarate ratio and flux directions. Especially, the ratios will not allow for the oxidative direction, neither in the cytosol nor in the mitochondria. Fumarase is a reversible reaction with an equilibrium constant *K_eq_* = 4.02. The measured (whole cell) Mal/Fum ratio is between 150 (t=52h) and 185 (t=73h, Table 3), which is far beyond the required thermodynamic driving force for this reaction. From thermodynamics it follows that fumarase operates in the direction of the oxidative TCA cycle if the mass action ratio Mal/Fum is lower than *K_eq_*. Based on this, a maximal mitochondrial malate concentration can be estimated. Assuming that all fumarate is mitochondrial (~0.5 µmol/g, equivalent to ~4.2 mmol/L_M_ ) a maximum value of 14.7 mmol/L_M_ is obtained for malate. This mitochondrial concentration corresponds to 1.8 µmol malate per g biomass, suggesting that the measured total amount of about 93 µmol/g biomass(S3) must be distributed very unequal, e.g. 90.9 µmol/g in the cytosol and 1.8 µmol/g in the mitochondria (~0.7%). Clearly, this calculation is based on several assumptions that require further validation, nevertheless reaction directions can only be fulfilled with an unequal distribution. Thus, we did take this distribution as starting point for the other Malate/Fumarate dependent carriers, and a significantly different distribution is obtained (see ). The big difference suggests, that not all carriers are at equilibrium and results obtained from the ^13^C based estimation outside the equilibrium considerations can be accepted.

The calculations are based on reported transport mechanisms. As essential first component, the equilibrium of the Phosphate/Proton symport is determined:

Phosphate dissociation has to be taken into account because of the different pH in the compartments:

p*K_a1_* 2.12

p*K_a2_* 7.21

p*K_a3_* 12.67

The transport equilibrium is reached when

Leading to:

$$\frac{\left(H_{2}PO_{4}^{-}\right) _{M}}{\left(H_{2}PO_{4}^{-}\right)_{C}}=10^{pH_{M}-pH_{C}}=3.16 \frac{10^{(pKa2-pH_{M})}\left(HPO_{4}^{2-}\right) _{M}}{10^{(pKa2-pH_{C})}\left(HPO_{4}^{2-}\right)_{C}}= \frac{\left(HPO_{4}^{2-}\right) _{M}}{\left(HPO_{4}^{2-}\right)_{C}}=\frac{10^{(pKa2-pH_{C})}}{10^{(pKa2-pH_{M})}} 10^{pH_{M}-pH_{C}}=10^{2pH_{M}-2pH_{C}}$$

There could be further processes involved in the total phosphate equilibrium, like the formation of a magnesium phosphate complex – nevertheless for the here applied study, the equilibrium of the -2 charged species is most relevant for the other carriers:

**Malate/Pi carrier:**

Malate p*K*_a1_ = 3.40, p*K*_a2_ = 5.20

At equilibrium:

$$\frac{\left(Mal^{2-}\right) _{M}}{\left(Mal^{2-}\right)_{C}}= \frac{\left(HPO_{4}^{2-}\right) _{M}}{\left(HPO_{4}^{2-}\right)_{C}}=10^{2pH_{M}-2pH_{C}}$$

Measured is the concentration of Malate in all states of dissociation. Mal^2-^ is replaced by the respective fraction of the total concentration at the mitochondrial / cytosolic pH:

The same transport mechanism can be assumed for succinate

p*K_a1_* = 4.2, p*K_a2_* = 5.6

For the citrate/malate carrier following stoichiometry is reported:

At equilibrium:

$$\frac{\left(HCit^{2-}\right) _{M}}{\left(HCit^{2-}\right)_{C}}=\frac{\left(Mal^{2-}\right) _{M}}{\left(Mal^{2-}\right)_{C}} =10^{2pH_{M}-2pH_{C}}$$

Replacing $\left(HCit^{2-}\right)$with the respective fraction of the total citrate (p*K_a1_* = 3.13, p*K_a2_* = 4.76, p*K_a3_* = 6.39):

$$\begin{matrix} \frac{\left(HCit^{2-}\right) _{M}}{\left(HCit^{2-}\right)_{C}} =10^{2pH_{M}-2pH_{C}}=\frac{\frac{10^{2pH_{M}}10^{pKa3}}{10^{3pH_{M}}+10^{2pH_{M}+pKa3}+10^{pH_{M}+pKa2+pKa3}+10^{pKa1+pKa2+pKa3}}\left(Cit\right)_{M}}{\frac{10^{2pH_{C}}10^{pKa3}}{10^{3pH_{C}}+10^{2pH_{C}+pKa3}+10^{pH_{C}+pKa2+pKa3}+10^{pKa1+pKa2+pKa3}}\left(Cit\right)_{C}} \\ \frac{\left(Cit^{x}\right) _{M}}{\left(Cit^{x}\right)_{C}}= \frac{ 10^{3pH_{M}}+10^{2pH_{M}+pKa3}+10^{pH_{M}+pKa2+pKa3}+10^{pKa1+pKa2+pKa3}}{ 10^{3pH_{C}}+10^{2pH_{C}+pKa3}+10^{pH_{C}+pKa2+pKa3}+10^{pKa1+pKa2+pKa3}} =25.5 \end{matrix}$$

Isocitrate/Citrate shuttle

Equilibrium ratio:

$$\frac{\left(Cit^{3-}\right) _{M}}{\left(Cit^{3-}\right)_{C}}=\frac{\left(iCit^{3-}\right) _{M}}{\left(iCit^{3-}\right)_{C}} =10^{2pH_{M}-2pH_{C}}\frac{10^{(pH_{M}-pKa3)}}{10^{(pH_{C}-pKa3)}}=10^{3pH_{M}-3pH_{C}}$$

Replacing $\left(iCit^{3-}\right)$with the respective fraction of the total iso-citrate (p*K_a1_* = 3.29, p*K_a2_* = 4.71, p*K_a3_* = 6.40):

$$\frac{\left(iCit^{x}\right) _{M}}{\left(iCit^{x}\right)_{C}}= 10^{3pH_{M}-3pH_{C}}\frac{10^{3pH_{C}} \left(10^{pH_{C}+pKa2+pKa3}+10^{pKa1+pKa2+pKa3}+10^{3pH_{C}}+10^{2pH_{C}+pKa3}\right)}{10^{3pH_{M}}\left(10^{pH_{M}+pKa2+pKa3}+10^{pKa1+pKa2+pKa3}+10^{3pH_{M}}+10^{2pH_{M}+pKa3}\right)}\frac{\left(Cit^{3-}\right)_{M}}{\left(Cit^{3-}\right)_{C}}$$

Citrate/aKG shuttle:

At equilibrium:

$$\frac{\left(HCit^{2-}\right) _{M}}{\left(HCit^{2-}\right)_{C}}=\frac{\left(aKG^{2-}\right) _{M}}{\left(aKG^{2-}\right)_{C}} =10^{2pH_{M}-2pH_{C}}$$

Replacing aKG^2-^ with aKG_tot_ (p*K_a1_* = 2.66, p*K_a2_* = 4.6):

$$\begin{matrix} \frac{\left(aKG^{2-}\right) _{M}}{\left(aKG^{2-}\right)_{C}} =\frac{\left(aKG\right) _{M}}{\left(aKG\right)_{C}}\frac{10^{2pH_{M}}\left(10^{2pH_{C}}+10^{pH_{C}+pKa2}+10^{pKa1+pKa2}\right)}{10^{2pH_{C}}\left(10^{2pH_{M}}+10^{pH_{M}+pKa2}+10^{pKa1+pKa2}\right)}=10^{2pH_{M}-2pH_{C}} \\ \frac{\left(aKG\right) _{M}}{\left(aKG\right)_{C}}=\frac{\left(10^{2pH_{C}}+10^{pH_{C}+pKa2}+10^{pKa1+pKa2}\right)}{\left(10^{2pH_{M}}+10^{pH_{M}+pKa2}+10^{pKa1+pKa2}\right)}=\text{ 9.96} \end{matrix}$$

Fumarate is transported using the succinate/fumarate shuttle:

At equilibrium:

$$\frac{\left(Fum^{2-}\right) _{M}}{\left(Fum^{2-}\right)_{C}}=\frac{\left(Succ^{2-}\right) _{M}}{\left(Succ^{2-}\right)_{C}} =10^{2pH_{M}-2pH_{C}}$$

Replacing $\left(Fum^{2-}\right)$with the respective fraction of the total fumarate (p*K_a1_* = 3.03, p*K_a2_* = 4.44):

The resulting equilibrium ratios are reported in of the main text.

**Table S4:** Thermodynamic consistency w.r.t. to estimated compartment specific concentrations and flux estimation direction at t=53h. For cytosol and mitochondria the reactions are displayed in direction of the flux estimation. For a consistent thermodynamic driving force, Q/Keq has to be below 1. Inconsistent ratios are marked with grey background. In case of the mitochondrial carriers the reaction is displayed as defined in the model, the reported K_eq_ reflects the equilibrium at the assumed pH (pH_mit_ = 7.3, pH_cyt_ = 6.8) and electrochemical potential difference (-180 mV).

| Reaction (flux) | $K_{eq}^{0'}$ | $Q$ | $Q/K_{eq}^{0'}$ |
| --- | --- | --- | --- |
| Cit -> iCit | 4.6E-02 | 7.5E-02 | 1.6E+00 |
| iCit + NAD -> aKG + CO_2_ + NADH | 1.1E-01 | 2.4E-04 | 2.1E-03 |
| aKG + ADP + NAD -> Succ + CO_2_ + NADH + ATP | 3.4E+04 | 2.4E-04 | 7.2E-09 |
| Succ + FAD -> Fum + FADH_2_ | 4.1E-09 | 5.2E-03 | 1.3E+06 |
| Fum -> Mal | 4.0E+00 | 6.5E-01 | 1.6E-01 |
| Mal + NAD -> OAA + NADH | 9.6E-06 | 4.1E-01 | 4.3E+04 |
| Reaction (flux) | $K_{eq}^{0'}$ | $Q$ | $Q/K_{eq}^{0'}$ |
| Fum + NADH -> Succ + NAD | 1.2E+11 | 7.82E+04 | 6.6E-07 |
| Mal -> Fum | 2.5E-01 | 1.31E-03 | 5.3E-03 |
| OAA + NADH -> Mal + NAD | 2.1E+05 | 1.37E+05 | 6.6E-01 |
| Reaction | $K_{eq}^{0'}$ | $Q$ | Est. flux direction |
| Fum_mit + Succ_cyt <> Fum_cyt + Succ_mit | 0.96 | 0.02 | bwd |
| Mal_mit + Pi_cyt <> Mal_cyt + Pi_mit | 0.32 | 28.95 | bwd |
| Cit_mit + Mal_cyt <> Cit_cyt + Mal_mit | 0.39 | 0.02 | fwd |
| iCit_mit + Cit_cyt <> iCit_cyt + Cit_mit | 1.00 | 3.05 | bwd |
| aKG_mit + Cit_cyt <> aKG_cyt + Cit_mit | 2.56 | 0.33 | fwd |
| pi_mit + succ_cyt  -> pi_cyt + succ_mit | 3.03 | 51.85 | bwd |

**Table S5:** Thermodynamic consistency w.r.t. to estimated compartment specific concentrations and flux estimation direction at t=73h. Inconsistencies are marked in grey.

| Mitochondria | | | |
| --- | --- | --- | --- |
| Reaction (flux) | $K_{eq}^{0'}$ | $Q$ | $Q/K_{eq}^{0'}$ |
| Cit -> iCit | 4.6E-02 | 1.2E-01 | 2.7E+00 |
| iCit + NAD -> aKG + CO2 + NADH | 1.1E-01 | 8.3E-05 | 7.4E-04 |
| aKG + ADP + NAD -> Succ + CO2 + NADH + ATP | 3.4E+04 | 3.6E-04 | 1.0E-08 |
| Succ + FAD -> Fum + FADH2 | 4.1E-09 | 3.5E-03 | 8.5E+05 |
| Fum -> Mal | 4.0E+00 | 4.6E-01 | 1.2E-01 |
| Mal + NAD -> OAA + NADH | 9.6E-06 | 3.4E-01 | 3.5E+04 |
| Cytosol | | | |
| Reaction (flux) | $K_{eq}^{0'}$ | $Q$ | $Q/K_{eq}^{0'}$ |
| Fum + NADH -> Succ + NAD | 1.2E+11 | 2.96E+04 | 2.5E-07 |
| Mal -> Fum | 2.5E-01 | 3.24E-03 | 1.3E-02 |
| OAA + NADH -> Mal + NAD | 2.1E+05 | 1.28E+05 | 6.1E-01 |
| Mitochondrial carrier | | | |
| Reaction | $K_{eq}^{0'}$ | $Q$ | Est. flux direction |
| Fum_mit + Succ_cyt <> Fum_cyt + Succ_mit | 0.96 | 0.10 | fwd |
| Mal_mit + Pi_cyt <> Mal_cyt + Pi_mit | 0.32 | 99.90 | bwd |
| Cit_mit + Mal_cyt <> Cit_cyt + Mal_mit | 0.39 | 0.01 | fwd |
| iCit_mit + Cit_cyt <> iCit_cyt + Cit_mit | 1.00 | 3.03 | bwd |
| aKG_mit + Cit_cyt <> aKG_cyt + Cit_mit | 2.56 | 0.33 | fwd |
| pi_mit + succ_cyt  -> pi_cyt + succ_mit | 3.03 | 19.54 | bwd |

**Table S6:** Measured mass isotopomer distribution during the first experiment (t=52h). Shown are the carbon enrichments, i.e. all values are corrected for isotopes of non-carbon skeleton atoms.

|  |  | Sampling time | | | | | |
| --- | --- | --- | --- | --- | --- | --- | --- |
| Metabolite | **Mass** | **0s** | **18s** | **64s** | **83s** | **177s** | **237s** |
| aKG | +0 | 93.42% | 91.58% | 85.54% | 86.23% | 82.27% | 81.15% |
|  | +1 | 5.55% | 7.55% | 13.84% | 12.85% | 16.60% | 17.89% |
|  | +2 | 0.21% | 0.42% | 0.36% | 0.75% | 0.82% | 0.84% |
|  | +3 | 0.47% | 0.08% | 0.03% | 0.06% | 0.16% | 0.03% |
|  | +4 | 0.15% | 0.13% | 0.02% | 0.01% | 0.01% | 0.04% |
|  | +5 | 0.19% | 0.23% | 0.21% | 0.10% | 0.14% | 0.05% |
| Asp | +0 | 95.04% | 95.55% | 79.48% | 78.77% | 76.91% | 73.53% |
|  | +1 | 5.63% | 1.76% | 6.04% | 6.05% | 7.21% | 8.71% |
|  | +2 | -1.12% | 2.57% | 13.41% | 14.34% | 15.06% | 16.95% |
|  | +3 | 0.28% | 0.03% | 1.06% | 0.58% | 0.46% | 0.62% |
|  | +4 | 0.18% | 0.08% | 0.02% | 0.26% | 0.36% | 0.19% |
| Cit | +0 | 94.22% | 93.48% | 92.70% | 89.06% | 94.24% | 92.16% |
|  | +1 | 5.21% | 4.85% | 5.05% | 5.63% | 3.77% | 2.52% |
|  | +2 | 0.90% | 1.06% | 0.98% | 6.55% | 2.19% | 5.75% |
|  | +3 | -0.32% | 0.33% | 0.56% | -1.16% | -0.22% | 0.21% |
|  | +4 | -0.03% | 0.09% | 0.93% | -0.65% | -0.35% | -0.78% |
|  | +5 | 0.01% | 0.23% | -0.21% | 0.58% | 0.40% | 0.05% |
|  | +6 | 0.01% | -0.04% | -0.02% | -0.01% | -0.02% | 0.09% |
| Fum | +0 | 94.25% | 79.29% | 68.54% | 70.14% | 64.85% | 75.69% |
|  | +1 | 4.97% | 6.52% | 5.08% | 4.57% | 6.17% | 5.39% |
|  | +2 | 0.72% | 14.18% | 23.82% | 25.33% | 25.92% | 17.81% |
|  | +3 | 0.00% | 0.06% | 1.56% | 0.12% | 2.44% | 0.11% |
|  | +4 | 0.06% | -0.05% | 0.99% | -0.16% | 0.62% | 1.00% |
| Glu | +0 | 96.14% | 94.71% | 90.94% | 90.31% | 88.63% | 81.89% |
|  | +1 | 3.79% | 5.64% | 8.20% | 9.34% | 11.03% | 17.15% |
|  | +2 | -0.38% | -0.42% | 0.61% | 0.03% | -0.26% | 0.71% |
|  | +3 | 0.05% | -0.18% | 0.49% | 0.26% | 0.58% | 0.31% |
|  | +4 | 0.46% | 0.19% | -0.24% | 0.05% | -0.03% | -0.12% |
|  | +5 | -0.06% | 0.06% | 0.01% | 0.02% | 0.05% | 0.06% |
| Glx | +0 | 89.42% | 85.35% | 84.32% | 88.12% | 86.17% | 88.38% |
|  | +1 | 9.49% | 12.78% | 12.86% | 10.39% | 11.53% | 8.67% |
|  | +2 | 1.09% | 1.87% | 2.82% | 1.49% | 2.30% | 2.95% |
| iCit | +0 | 94.21% | 93.29% | 83.58% | 83.10% | 79.78% | 81.44% |
|  | +1 | 5.80% | 5.35% | 6.84% | 5.89% | 8.77% | 9.18% |
|  | +2 | -0.01% | 1.32% | 9.04% | 10.02% | 10.64% | 8.68% |
|  | +3 | -0.01% | -0.01% | 0.63% | 0.82% | 0.77% | 0.66% |
|  | +4 | 0.03% | 0.05% | -0.03% | 0.21% | 0.03% | 0.07% |
|  | +5 | -0.02% | -0.05% | -0.06% | -0.06% | -0.06% | -0.02% |
|  | +6 | 0.00% | 0.05% | 0.01% | 0.01% | 0.07% | 0.01% |

| Mal | +0 | 95.98% | 95.12% | 94.67% | 95.20% | 93.70% | 92.26% |
| --- | --- | --- | --- | --- | --- | --- | --- |
|  | +1 | 3.94% | 3.64% | 3.46% | 3.89% | 4.96% | 5.56% |
|  | +2 | 0.08% | 1.28% | 1.33% | 0.85% | 1.10% | 2.04% |
|  | +3 | 0.02% | -0.06% | 0.38% | 0.10% | 0.12% | 0.12% |
|  | +4 | -0.01% | 0.02% | 0.15% | -0.03% | 0.11% | 0.02% |
| Oaa | +0 | 89.54% | 80.21% | 76.34% | 79.64% | 81.00% | 75.94% |
|  | +1 | 6.11% | 8.19% | 9.13% | 7.65% | 9.56% | 8.72% |
|  | +2 | 3.40% | 7.92% | 12.10% | 10.66% | 7.26% | 13.13% |
|  | +3 | 0.42% | 3.10% | 0.54% | 1.30% | 0.87% | 1.83% |
|  | +4 | 0.53% | 0.58% | 1.88% | 0.75% | 1.32% | 0.37% |
| Succ | +0 |  | 22.99% | 21.05% | 20.54% | 20.06% | 21.24% |
|  | +1 |  | 1.58% | 1.51% | 1.45% | 1.47% | 1.53% |
|  | +2 |  | 73.65% | 75.73% | 76.35% | 76.80% | 75.56% |
|  | +3 |  | 1.79% | 1.77% | 1.70% | 1.71% | 1.72% |
|  | +4 |  | 0.00% | -0.06% | -0.03% | -0.05% | -0.06% |
| EC Mal | +0 |  | 95.91% | 95.68% | 95.54% | 95.50% | 95.15% |
|  | +1 |  | 3.91% | 4.07% | 4.05% | 4.09% | 4.14% |
|  | +2 |  | 0.14% | 0.24% | 0.42% | 0.33% | 0.74% |
|  | +3 |  | 0.03% | 0.00% | -0.01% | 0.06% | -0.03% |
|  | +4 |  | 0.01% | 0.01% | 0.01% | 0.02% | 0.00% |
| EC Succ | +0 | 95.68% | 19.36% | 18.40% | 19.18% | 19.12% | 18.67% |
|  | +1 | 4.28% | 1.40% | 1.36% | 1.39% | 1.40% | 1.39% |
|  | +2 | 0.03% | 77.42% | 78.42% | 77.62% | 77.63% | 78.13% |
|  | +3 | 0.01% | 1.79% | 1.77% | 1.78% | 1.78% | 1.76% |
|  | +4 | 0.00% | 0.04% | 0.05% | 0.04% | 0.07% | 0.05% |

**Table S7:** Measured mass isotopomer distribution during the second experiment (t=73h). Shown are the carbon enrichments, i.e. all values are corrected for isotopes of non-carbon skeleton atoms.

|  |  | Sampling time | | | | | |
| --- | --- | --- | --- | --- | --- | --- | --- |
| Metabolite | Mass | 0s | 26s | 64s | 83s | 177s | 237s |
| aKG | +0 | 94.31% | 92.98% | 81.10% | 84.55% | 76.67% | 76.71% |
|  | +1 | 5.17% | 6.83% | 12.97% | 14.63% | 20.73% | 21.78% |
|  | +2 | 0.22% | 0.17% | 1.68% | 0.65% | 1.78% | 1.32% |
|  | +3 | 0.12% | -0.03% | 0.68% | 0.06% | 0.06% | 0.05% |
|  | +4 | 0.02% | 0.01% | 1.71% | 0.04% | 0.20% | 0.05% |
|  | +5 | 0.16% | 0.05% | 1.87% | 0.06% | 0.56% | 0.09% |
| Asp | +0 | 94.79% | 96.99% | 95.45% | 83.69% | 77.69% | 81.32% |
|  | +1 | 5.06% | 3.25% | 2.81% | 1.87% | 8.35% | 8.28% |
|  | +2 | -0.16% | -0.69% | 1.46% | 11.44% | 13.27% | 9.40% |
|  | +3 | 0.01% | 0.51% | 0.47% | 2.71% | 0.38% | 0.69% |
|  | +4 | 0.30% | -0.05% | -0.19% | 0.29% | 0.31% | 0.31% |
| Cit | +0 | 93.97% | 92.55% | 94.25% | 88.12% | 92.63% | 94.77% |
|  | +1 | 4.91% | 6.72% | 3.53% | 8.04% | 6.92% | 3.63% |
|  | +2 | 1.24% | 0.98% | 1.45% | 2.42% | -0.43% | 0.60% |
|  | +3 | -0.06% | -0.21% | 0.33% | 0.87% | 0.40% | 0.23% |
|  | +4 | -0.14% | -0.33% | 0.61% | 0.68% | 0.67% | 0.99% |
|  | +5 | 0.01% | 0.23% | -0.15% | -0.10% | -0.16% | -0.19% |
|  | +6 | 0.07% | 0.05% | -0.01% | -0.02% | -0.02% | -0.03% |
| Fum | +0 | 93.53% | 85.98% | 66.47% | 71.28% | 70.18% | 69.23% |
|  | +1 | 4.80% | 4.85% | 7.60% | 4.16% | 4.50% | 8.95% |
|  | +2 | 1.44% | 8.69% | 22.64% | 22.84% | 23.78% | 21.17% |
|  | +3 | 0.06% | 0.53% | 2.37% | 0.61% | 0.20% | 0.58% |
|  | +4 | 0.17% | -0.06% | 0.92% | 1.11% | 1.34% | 0.07% |
| Glu | +0 | 95.11% | 95.46% | 95.05% | 92.25% | 87.39% | 80.58% |
|  | +1 | 4.56% | 4.62% | 5.47% | 6.45% | 12.97% | 19.01% |
|  | +2 | 0.59% | -0.12% | -0.74% | 0.60% | -0.62% | 0.24% |
|  | +3 | -0.35% | -0.07% | 0.09% | 0.26% | 0.06% | 0.11% |
|  | +4 | 0.01% | 0.04% | 0.09% | 0.27% | 0.24% | 0.05% |
|  | +5 | 0.09% | 0.07% | 0.03% | 0.19% | -0.03% | 0.01% |
| Glx | +0 | 94.40% | 84.64% | 79.54% | 74.13% | 81.52% | 83.53% |
|  | +1 | 4.87% | 13.05% | 18.84% | 22.60% | 15.08% | 13.68% |
|  | +2 | 0.73% | 2.31% | 1.62% | 3.28% | 3.40% | 2.80% |
| iCit | +0 | 94.29% | 93.68% | 86.77% | 83.77% | 80.27% | 85.93% |
|  | +1 | 5.41% | 5.58% | 6.64% | 6.94% | 7.79% | 6.73% |
|  | +2 | 0.17% | 0.53% | 6.23% | 8.25% | 10.82% | 6.64% |
|  | +3 | 0.09% | 0.19% | 0.33% | 1.00% | 0.96% | 0.68% |
|  | +4 | 0.04% | 0.03% | 0.06% | 0.01% | 0.13% | -0.05% |
|  | +5 | 0.00% | -0.02% | -0.01% | 0.01% | 0.02% | 0.04% |
|  | +6 | 0.01% | 0.01% | -0.03% | 0.03% | 0.02% | 0.02% |

| Mal | +0 | 95.89% | 95.69% | 95.59% | 95.60% | 95.31% | 95.56% |
| --- | --- | --- | --- | --- | --- | --- | --- |
|  | +1 | 4.05% | 3.76% | 4.09% | 3.72% | 4.17% | 3.76% |
|  | +2 | 0.04% | 0.59% | 0.41% | 0.57% | 0.41% | 0.68% |
|  | +3 | 0.02% | -0.08% | -0.12% | -0.01% | 0.09% | -0.01% |
|  | +4 | 0.00% | 0.04% | 0.03% | 0.12% | 0.02% | 0.01% |
| Oaa | +0 | 73.85% | 53.38% | 76.77% | 74.72% | 64.40% | 87.50% |
|  | +1 | 17.32% | 31.69% | 13.57% | 14.99% | 25.51% | 7.13% |
|  | +2 | 6.34% | 11.09% | 8.04% | 7.21% | 8.63% | 4.33% |
|  | +3 | 1.96% | 1.39% | 0.34% | 1.95% | 0.60% | 0.61% |
|  | +4 | 0.53% | 2.46% | 1.28% | 1.13% | 0.87% | 0.44% |
| Succ | +0 | 95.70% | 21.58% | 18.74% | 18.71% | 19.09% | 19.38% |
|  | +1 | 4.21% | 1.55% | 1.47% | 1.40% | 1.45% | 1.43% |
|  | +2 | 0.09% | 75.17% | 78.06% | 78.27% | 77.78% | 77.48% |
|  | +3 | 0.00% | 1.78% | 1.79% | 1.74% | 1.76% | 1.82% |
|  | +4 | 0.00% | -0.09% | -0.07% | -0.12% | -0.08% | -0.11% |
| EC Mal | +0 | 96.30% | 95.90% | 95.97% | 95.99% | 95.73% | 95.88% |
|  | +1 | 3.75% | 3.99% | 3.95% | 3.87% | 4.07% | 3.89% |
|  | +2 | -0.06% | 0.11% | 0.03% | 0.11% | 0.15% | 0.22% |
|  | +3 | 0.02% | 0.00% | 0.04% | 0.04% | 0.04% | 0.00% |
|  | +4 | 0.00% | 0.01% | 0.00% | -0.01% | 0.01% | 0.02% |
| EC Succ | +0 | 95.24% | 16.52% | 16.50% | 16.62% | 16.39% | 16.46% |
|  | +1 | 4.10% | 1.31% | 1.28% | 1.29% | 1.31% | 1.30% |
|  | +2 | 0.65% | 80.27% | 80.30% | 80.15% | 80.38% | 80.33% |
|  | +3 | 0.02% | 1.83% | 1.84% | 1.86% | 1.86% | 1.86% |
|  | +4 | -0.01% | 0.07% | 0.07% | 0.08% | 0.05% | 0.06% |
